# A fibril-scale visco-hyperelastic model for the mechanics of vocal-fold tissues

**DOI:** 10.3389/fbioe.2025.1670567

**Published:** 2026-01-05

**Authors:** Alberto Terzolo, Lucie Bailly, Laurent Orgéas

**Affiliations:** University Grenoble Alpes, CNRS, Grenoble INP, 3SR, Grenoble, France

**Keywords:** vocal folds, fibril, 3D microstructure, multiscale mechanical modeling, viscoelasticity, multi-axial loadings, SAOS, LAOS

## Abstract

**Introduction:**

Modeling the mechanics of human vocal folds during phonation is a challenging task. This is partly due to the mechanics of their soft and highly anisotropic fibrous tissues, which undergoes finite strains with both elasticity and strain-rate sensitivity.

**Methods:**

We propose a visco-hyperelastic micro-mechanical model capable of predicting the complex cyclic response of the vocal-fold fibrous tissues based on their histo-mechanical properties. For that purpose, we start from the hyperelastic micro-mechanical model proposed by Terzolo et al., *J. Mech. Behav. Biomed. Mater.* 128:105118 (2022). We include in the model non-linear viscoelastic contributions at the fibril scale to account for the dissipative and time-dependent response of vocal-fold tissues.

**Results and Discussion:**

The relevance of the model is demonstrated and discussed through comparison with a comprehensive set of reference experimental data, within a wide range of loading modes, strains, and strain rates: cyclic and multi-axial loadings at finite strains (tension, compression and shear), along with small-amplitude oscillatory shear (SAOS) and large-amplitude oscillatory shear (LAOS) from low to high frequencies. This study elucidates how the viscoelasticity of vocal-fold tissues can result from combined time-dependent micro-mechanisms, such as the kinematics and the deformation of their fibril bundles, along with the mechanical interactions likely to develop among fibrils and the surrounding amorphous matrix.

## 1 Introduction

**FIGURE 1:**
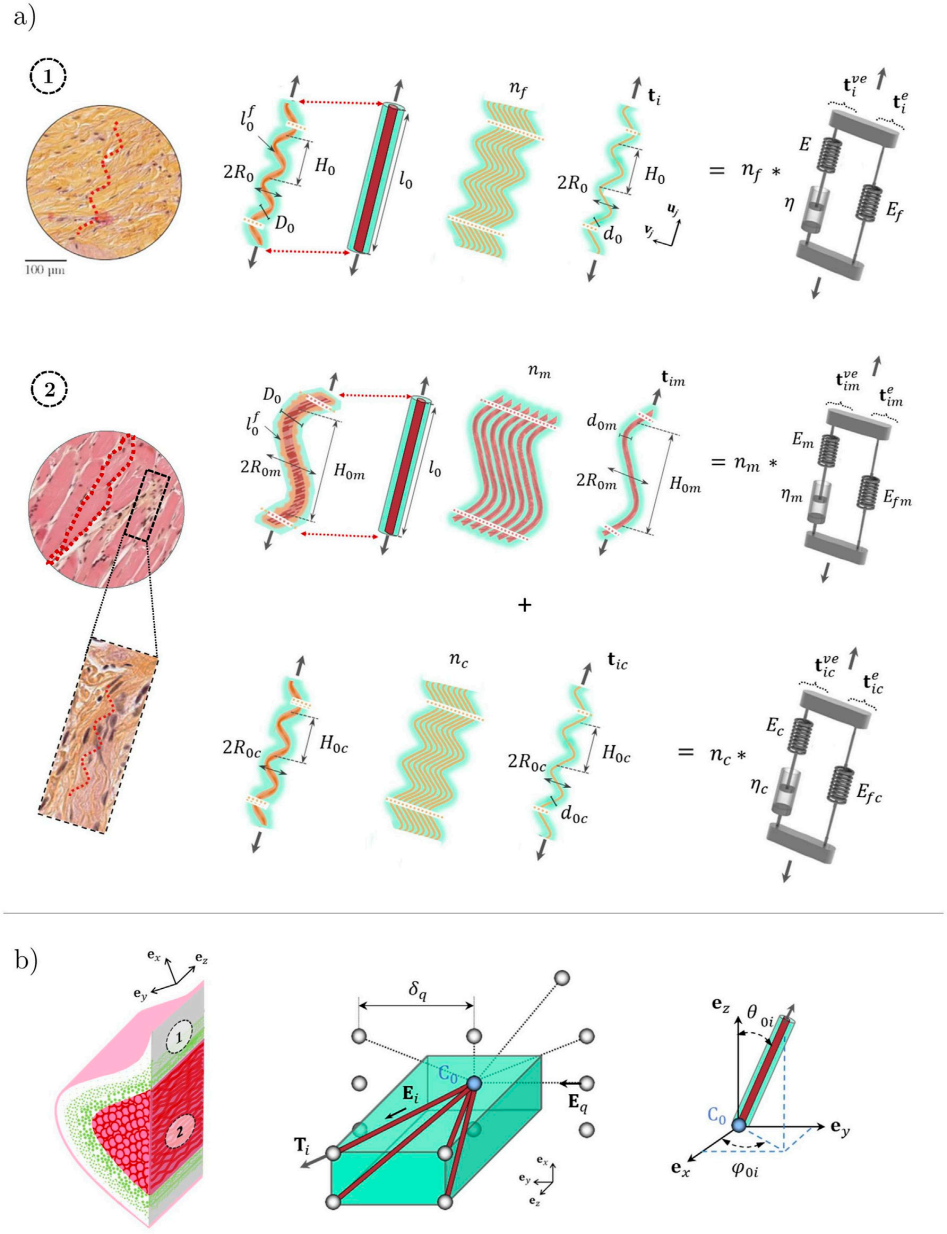
Idealization of the vocal-fold layers. **(a)** The *lamina propria* (respectively the *vocalis*), displayed on microscopic image 1 (respectively 2), is considered a network of (*orange*) collagen fibrils [respectively (*pink*) myofibrils and (orange) collagen fibrils] embedded into a gel-like matrix. Fibrils are self-assembled as collagen fibril bundles (respectively myofibrils surrounded by a sheath of collagen fibrils). Each fibril (and its interaction with its neighboring) behaves as a non-linear visco-hyperelastic Zener model. **(b)** The fiber bundle microstructure of each layer is considered a periodic network of four orientated fiber bundles (*brown*) connected at one node ${\text{C}}_{0}$ (*blue*) embedded in a soft isotropic matrix (*green*). The dotted lines illustrate the five possible steric interactions of ${\text{C}}_{0}$ with the neighboring nodes. *Source:* Adapted from Terzolo et al. (2022).

Human vocal folds are soft laryngeal structures with remarkable mechanical properties. During phonation, the vocal folds deform under the action of pulmonary airflow and laryngeal motions, sustaining vibrations in a wide range of amplitudes, frequencies (from 50 Hz to more than 1,500 Hz), and degrees of collisions. These multiple configurations involve complex and coupled multi-axial mechanical stresses (in tension, compression, and shear) that the tissues can withstand upon finite strains at various strain rates (Miri, 2014; Vampola et al., 2016). These properties are inherited from the composite and hierarchical structure of the vocal folds and surrounding laryngeal muscles. More specifically, the vocal folds are made up of two main load-bearing layers: the *lamina propria*, *i.e.*, a loose connective tissue, and the *vocalis* muscle. Both layers are composed of networks of collagen, elastin, or skeletal muscle microfibrils, embedded in a soft hydrogel-like matrix (Figure 1, Hirano, 1974, Benboujja and Hartnick, 2021, Ferri-Angulo et al., 2023). However, to date, our knowledge is still insufficient to understand the relationship between the fibril-scale architecture of the vocal folds and their macroscale (tissue-scale) time-dependent performance.

This is mainly due to the difficulty of characterizing vocal-fold mechanics at high physiological strain rates. Although recent progress has been made in time-resolved 3D micro-imaging of fast-vibrating structures (Klos et al., 2024), to date, the characterization of the mechanical behavior of vocal-fold tissues at high frequencies (*e.g.*, from 100 Hz to 1 kHz) is still limited to the larynx or vocal-fold scale. High-speed videostroboscopy, used in clinical voice assessment, enabled the quantification of the time-decay of vocal-fold vibrations at phonation offset (DeJonckere and Lebacq, 2020; Radolf et al., 2022), and of their resonance properties obtained through external excitation of the larynx (Švec et al., 2000). Such *in vivo* approaches allowed the measurement of an average damping ratio ζ≈ 0.07–0.20, describing the dissipation of stored energy in oscillations for frequencies between 100 and 200 Hz (Švec et al., 2000; DeJonckere and Lebacq, 2020; Radolf et al., 2022), which partly arises from the viscoelastic behavior of the tissues. The time-dependent mechanical properties of vocal-fold tissues have also been demonstrated *ex vivo* through numerous phenomena, including strain-rate sensitivity of stress–strain behavior, creep, stress relaxation, stress hysteresis, and related accommodation upon cycling, with the magnitude of the hysteresis loop dependent on the strain rate (Kelleher et al., 2013a; Chan and Titze, 1999; 2000; Chan, 2004; Klemuk and Titze, 2004; Titze et al., 2004; Chan and Rodriguez, 2008; Miri et al., 2014; Chan, 2018; Cochereau et al., 2020). The viscoelastic properties of excised *lamina propria* samples were mostly studied using standard shear dynamic mechanical analysis (DMA), also called small-amplitude oscillatory shear (SAOS), *i.e.*, within the linear regime (Chan and Titze, 1999; Chan and Titze, 2000; Chan, 2004; Klemuk and Titze, 2004; Titze et al., 2004; Chan and Rodriguez, 2008). Such works allowed to characterize the shear storage $G^{\prime }$ and loss $G^{\prime \prime }$ moduli of the vocal-fold “cover” (*i.e.*, the superficial sublayer of the *lamina propria* combined with the *epithelium* that covers it) for excitation frequencies f up to 250 Hz. Therefore, these dynamic moduli increase (respectively decrease) with the applied frequency (respectively strain), while the loss factor [ $\tan\delta =\zeta^{⋆}=G^{\prime \prime }/G^{\prime }$ (Dashatan et al., 2023; Koruk and Rajagopal, 2024)] decreases monotonically with frequency, down to a mean value of 0.73 for f within 100–250 Hz (Chan and Rodriguez, 2008). Such experiments were recently extended to large-amplitude oscillatory shear (LAOS), showing that *lamina propria* sublayers experience intercycle strain softening during oscillatory strain sweeps, intracycle strain stiffening, shear thinning while increasing the shear rate, and complex stress hysteresis that depends on the applied strain and strain rates (Chan, 2018).

To better analyze these data and unveil the underlying mechanisms, several theoretical approaches were adopted. Some phenomenological approaches were first developed (Zhang et al., 2006; Zhang et al., 2007; Zhang et al., 2009). However, the constitutive parameters of these models can hardly be related to relevant histological descriptors of the vocal tissues. Since 2010, a few authors have purposely proposed micro-mechanical models, including the architecture of vocal tissues, to provide new insights into voice biomechanics. Two modeling routes have been adopted:

(i) Poroelastic formulations have been developed to describe the fluid/solid phases of vocal tissues and to predict their dynamics (Miri et al., 2014; Tao et al., 2009; Scholp et al., 2020). However, such approaches rely on parameters for which experimental measurements (*e.g.*, permeability and *in situ* observations of fluid dynamics) are still lacking.

(ii) Other authors have idealized the architecture of the fibrous networks of the *lamina propria* and the *vocalis* (*e.g.*, using structural descriptors such as the fibril volume fraction, diameter, and preferred orientations) to derive their mechanical contribution from microstructural and micro-mechanical measurements (Miri et al., 2013; Kelleher et al., 2013b; Terzolo et al., 2022). This enabled the identification of the progressive elongation and reorientation of collagen fibrils and myofibrils, along with mechanical interactions between micro-constituents, which modulate the non-linear and anisotropic mechanics of vocal tissues (Terzolo et al., 2022). However, these micro-mechanical formulations have been developed within a general hyperelastic framework, thus neglecting the important dissipative and time-dependent mechanisms likely to develop during the vibrations of vocal tissues.

Therefore, this work aims to provide a multiscale mechanical model capable of reproducing the non-linear macroscopic visco-hyperelastic mechanical behavior of the vocal fold layers (*i.e.*, *lamina propria* and *vocalis*) across a range of frequencies and strains, based on the knowledge of their architecture and mechanics at the fibril scale. To achieve this, we introduce microstructural time-dependent effects to the hyperelastic formulation developed by Terzolo et al. (2022). Based on histological and biomechanical data available in the literature, covering a wide range of loading modes, strain levels, and rates, the relevance of the model for predicting the time-dependent, multiscale mechanics of the vocal-fold layers is highlighted and discussed.

## 2 Formulation of the micro-mechanical model

### 2.1 Structural assumptions

The structural assumptions of the model are identical to those reported by Terzolo et al. (2022). In brief, both the *lamina propria* and the *vocalis* are considered incompressible composite materials made of a gel-like matrix (composed of cells, elastin, and ground substance for the *lamina propria*; of elastin, proteoglycans, and glycoproteins for the *vocalis*) reinforced by a network of connected and oriented fibril bundles (Figure 1):

• For the *lamina propria* (Figure 1a, case ①), each fibril bundle is considered an assembly of parallel collagen fibrils with an initial diameter $d_{0}$ , length $\ell_{o}^{f}$ , and tortuosity $\xi_{0}=\ell_{0}^{f}/\ell_{0}$ , $\ell_{0}$ being their initial chord length. They are characterized by a waviness of approximately 10 monomodal sinusoids between nodes, with a wave amplitude $R_{0}$ and a spatial periodicity $H_{0}$ , so that $\ell_{0}\approx 10\;H_{0}$ at rest.

• For the *vocalis* (Figure 1a, case ②), each fibril bundle is considered an assembly of parallel myofibrils (with an initial diameter $d_{0m}$ , tortuosity $\xi_{0m}$ , wave amplitude $R_{0m}$ , spatial periodicity $H_{0m}$ , and chord length $\ell_{0}\approx 10H_{0m}$ ), surrounded by a sheath of collagen fibrils (with an initial diameter $d_{0c}$ , tortuosity $\xi_{0c}$ , wave amplitude $R_{0c}$ , and spatial periodicity $H_{0c}$ ).

• The fibrous architecture of the *lamina propria* exhibits a collagen fibril content Φ (leading to $n_{f}$ collagen fibrils in Figure 1a), whereas the *vocalis* displays a collagen fibril content $\Phi_{c}$ and a myofibril content $\Phi_{m}$ (leading to ${\text{n}}_{c}$ collagen fibrils and ${\text{n}}_{m}$ myofibrils). Both tissues are idealized as networks of connected fibril bundles. These networks are built from the periodic repetition of a representative elementary volume (REV), composed of four fibril bundles connected to a central node ${\text{C}}_{0}$ , and to four nodes ${\text{C}}_{i}$ of the corresponding neighboring REVs at their extremities (Figure 1b). At rest, each fibril bundle i is also characterized by its initial mean orientation $E_{i}$ , as depicted in Figure 1b. This set of orientation directors introduces structural anisotropy. The distances between node ${\text{C}}_{0}$ and its unconnected neighbors ${\text{C}}_{q}$ (see dotted lines in Figure 1b), *i.e.*, along the initial directions $E_{q}=C_{0}C_{q}/\|C_{0}C_{q}\|$ , are noted $\delta_{q}$ .

### 2.2 Micro-mechanical assumptions

*Kinematics*: When subjected to a macroscopic transformation gradient F and a macroscopic velocity gradient L , the tissue REVs deform from their initial configuration to a deformed configuration. As a consequence, fibril bundles (un)fold so that their chord length is $\ell_{i}=\ell_{0}\|F\cdot E_{i}\|$ in the deformed configuration, *i.e.*, with a tensile stretch and strain $\lambda_{i}$ = $\ell_{i}/\ell_{0}$ and $\varepsilon_{i}$ = $\ln\lambda_{i}$ , respectively. This process occurs at a tensile strain rate ${\dot{\varepsilon }}_{i}=e_{i}\cdot L\cdot e_{i}$ . Moreover, fibril bundles also rotate so that their current mean orientation directors become $e_{i}=F\cdot E_{i}/\|F\cdot E_{i}\|$ in the deformed configuration, thus introducing a strain-induced change in structural anisotropy. Finally, the rotation and the deformation of fibril bundles are not free and are hindered by steric effects between bundles. Steric effects are captured by restraining the motion of the node ${\text{C}}_{0}$ with respect to its unconnected neighbors ${\text{C}}_{q}$ . These restrictions occur along $e_{q}=F\cdot E_{q}/\|F\cdot E_{q}\|$ at a strain rate ${\dot{\varepsilon }}_{q}=e_{q}\cdot L\cdot e_{q}$ (see dotted lines in Figure 1b), once the distance $\delta_{q}$ between ${\text{C}}_{0}$ and the neighboring nodes ${\text{C}}_{q}$ exceeds a critical distance $\delta_{c}$ , *i.e.*, below a contact strain $\varepsilon_{q}$ = $\ln\left(\delta_{q}/\delta_{c}\right)$ .

*Mechanics of the matrix*: Regardless of the considered tissue, the mechanics of their matrix is modeled as an incompressible hyperelastic neo-Hookean medium, with a strain energy function $W=0.5\;\mu \left(1-\Phi \right)\left(tr\left(F\cdot F^{T}\right)-3\right)$ , which involves the shear modulus μ of the matrix.

*Mechanics of the fibrils*: The stretch (or the compression) of each fibril of a bundle i generates a non-linear fibril reaction force. This force is noted as $t_{i}=t_{i}e_{i}$ for the collagen fibrils of the *lamina propria*, and $t_{im}=t_{im}e_{i}$ and $t_{ic}=t_{ic}e_{i}$ for the collagen fibrils and the myofibrils of the *vocalis*, respectively. To mimic both the non-linear elasticity observed during the tension-compression of collagen fibrils and their time-dependent response, the following decomposition of the reaction force is proposed for the *lamina propria* (similar decompositions are proposed for $t_{im}$ and $t_{ic}$ in the case of the *vocalis*):

$$t_{i}=t_{i}^{e}+t_{i}^{ve},$$ (1)

where $t_{i}^{e}$ represents the (non-linear elastic) “neutral” response of the considered fibril, *i.e.*, when the system attains its “relaxed” configuration. The expression proposed by Terzolo et al. (2022) is used: it provides relevant estimate of the unfolding of fibrils while accounting for their dimension (diameter $d_{0}$ , chord length $\ell_{0}$ , and tortuosity $\xi_{0}$ ) and mechanical properties (elastic modulus $E_{f}$ ). Thus, $t_{i}^{e}$ is an hyperelastic function of $\varepsilon_{i}$ :

$$t_{i}^{e}=\frac{\pi d_{0}^{2}}{4}\left[E_{\mathrm{eq0}}\varepsilon_{i}+\frac{E_{f}-E_{\mathrm{eq0}}}{2}\left(\varepsilon_{i}+\sqrt{{\left(\varepsilon_{i}-\ln\xi_{0}\right)}^{2}+\alpha^{2}}-\sqrt{\ln^{2}\xi_{0}+\alpha^{2}}\right)\right],$$ (2)

when the fibril is stretched; only the first term of the bracket is kept when the fibril is compressed. This expression involves a curvature parameter α that ensures a proper transition between bending- and stretching-dominated regimes during fibril unfolding. In addition, the initial apparent modulus of the fibril in the folded configuration $E_{\mathrm{eq0}}=E_{f}\left\langle \cos\beta_{0}\right\rangle /\;\left[\left\langle \cos^{2}\beta_{0}\right\rangle +16\left\langle v^{2}\right\rangle /d_{0}^{2}\right]$ (with $\left\langle \cdot \right\rangle =\frac{1}{\ell_{0}}\int_{0}^{\ell_{0}}\cdot \;du$ , $\left\langle v^{2}\right\rangle =R_{0}^{2}/2$ , and $\beta_{0}=\;\arctan\left(2\pi \frac{R_{0}}{H_{0}}\cos\frac{2\pi }{H_{0}}u\right)$ ) is estimated from the literature (Potier-Ferry and Siad, 1992).

Moreover, in Equation 1, $t_{i}^{ve}$ represents time-dependent phenomena, including those related to the fibril deformation itself, the fibril interactions with the other fibrils and the surrounding gel-like matrix. These molecular-scale mechanisms exhibit characteristic relaxation times (Gautieri et al., 2012; Miri et al., 2013) that are not captured by the hyperelastic formulation proposed for $t_{i}^{e}$ in Equation 2. A fine quantification of these transient complex processes would require molecular-scale analyses based on statistical physics or numerical simulation using molecular dynamics approaches (Gautieri et al., 2011; Bantawa et al., 2023). Here, as a first approximation, we consider a simple approach at the scale of the fibrils to account for them. We assume that the aforementioned time-dependent phenomena can be reproduced using a non-linear viscoelastic Maxwell model (Equation 3), as schematized in Figure 1a:

$${\dot{t}}_{i}^{ve}+\frac{E}{\eta }t_{i}^{ve}=\pi E\frac{d_{0}^{2}}{4}{\dot{\varepsilon }}_{i},\;$$ (3)

where E and η are the elastic modulus and the viscosity of the Maxwell model, respectively. As vocal-fold tissues exhibit several relaxation times over a wide range of strain rates (Chan and Titze, 1999; 2000; Chan and Rodriguez, 2008; Chan, 2018), it is necessary to include these effects in Equation 3. For example, SAOS studies (Chan and Rodriguez, 2008) performed on *lamina propria* samples report a Carreau-like evolution of the complex viscosity with the shear rate, *i.e.*, with a Newtonian plateau at low shear rates and shear-thinning evolution at high shear rates. These aspects are taken into account by assuming that the viscosity η is a non-linear Carreau function of the viscous strain rate, as shown in Equation 4:

$$\eta =\eta_{0}{\left(1+{\left(\frac{\dot{\varepsilon_{i}}-\frac{{4\dot{t}}_{i}^{ve}}{\pi Ed_{0}^{2}}}{{\dot{\varepsilon }}_{0}}\right)}^{2}\right)}^{\frac{n-1}{2}},$$ (4)

where $\eta_{0}$ is the viscosity of the Newtonian regime, ${\dot{\varepsilon }}_{0}$ is the strain-rate transition between the Newtonian regime and the shear-thinning regime, and n is the power-law index driving thinning effects at high strain rates. Expressions similar to Equations 2–4 are proposed for the *vocalis*, further assuming that $E_{0c}=E_{0m}=E$ , $\eta_{0c}=\eta_{0m}=\eta_{0}$ , ${\dot{\varepsilon }}_{c0}={\dot{\varepsilon }}_{m0}={\dot{\varepsilon }}_{0}$ , and $n_{c}=n_{m}=n$ .

*Steric interactions between fibril bundle*: For both tissues, once the distance $\delta_{q}$ between the node ${\text{C}}_{0}$ and the neighboring nodes ${\text{C}}_{q}$ exceeds a critical distance $\delta_{c}$ , *i.e.*, below a contact strain $\varepsilon_{q}$ = $\ln\left(\delta_{q}/\delta_{c}\right)$ , steric interactions occur via reaction forces $R_{q}=R_{q}e_{q}$ . A decomposition similar to Equation 1 is proposed to account for non-linear visco-hyperelastic effects (Equation 5):

$$R_{q}=R_{q}^{e}+R_{q}^{ve},$$ (5)

where the hyperelastic term $R_{q}^{e}$ was proposed by Terzolo et al. (2022), as presented in Equation 6:

$$R_{q}^{e}=\beta \;H\left(\varepsilon_{q}\right)\;\varepsilon_{q}^{\kappa },$$ (6)

where H is the Heaviside function and β and κ are interaction parameters. To account for non-linear viscoelastic interactions, $R_{q}^{ve}$ is derived from the following non-linear Maxwell equation (Equation 7):

$${\dot{R}}_{q}^{ve}+\frac{E^{\prime }}{\eta^{\prime }}R_{q}^{ve}=E^{\prime }d_{0}{\dot{\varepsilon }}_{q}.$$ (7)

In analogy with Equation 4, the viscosity $\eta^{\prime }$ is assumed to be a Carreau function of the corresponding steric strain rate (Equation 8):

$$\eta^{\prime }=\eta_{0}^{\prime }{\left(1+{\left(\frac{{\dot{\varepsilon }}_{q}-\frac{{4\dot{R}}_{q}^{ve}}{\pi E^{\prime }d_{0}^{2}}}{\dot{\varepsilon_{0}^{\prime }}}\right)}^{2}\right)}^{\frac{n-1}{2}},$$ (8)

where $\eta_{0}^{\prime }$ is the viscosity of the Newtonian regime and ${\dot{\varepsilon }}_{0}^{\prime }$ is the transition strain rate between the Newtonian and thinning regimes.

### 2.3 Upscaling formulation: from micro- to macroscale mechanics

Given the structural and micro-mechanical features mentioned above, regardless of the tissue concerned, the macroscopic Cauchy stress tensor σ can be written as follows (Equation 9):

$$\sigma =-p\delta +\sigma_{m}+\sigma_{f}+\sigma_{s},$$ (9)

where p is the incompressibility pressure, δ is the identity tensor, $\sigma_{m}=F\cdot {\left(\partial W/\partial F\right)}^{T}$ is the stress contribution of the matrix, and $\sigma_{f}$ and $\sigma_{s}$ represent the stress contributions due to the (un)folding of fibrils and their steric interactions, respectively. Thus, Equations 10, 11 can be obtained as follows:

$$\sigma_{f}=\frac{\Phi }{\pi d_{0}^{2}\xi_{0}}\overset{4}{\underset{i=1}{\sum }}t_{i}\lambda_{i}\;e_{i}⊗e_{i}$$ (10)

and

$$\sigma_{s}=\frac{\Phi }{\pi d_{0}^{2}\xi_{0}}\overset{5}{\underset{q=1}{\sum }}R_{q}\delta_{q}^{*}\;e_{q}⊗e_{q}$$ (11)

for the *lamina propria*, where $\delta_{q}^{*}=\delta_{q}/\ell_{0}$ . Equations 12, 13 can be obtained as follows:

$$\sigma_{f}=\frac{\Phi_{c}}{\pi d_{0c}^{2}\xi_{0c}}\overset{4}{\underset{i=1}{\sum }}t_{ic}\lambda_{i}\;e_{i}⊗e_{i}+\frac{\Phi_{m}}{\pi d_{0m}^{2}\xi_{0m}}\overset{4}{\underset{i=1}{\sum }}t_{im}\lambda_{i}\;e_{i}⊗e_{i}$$ (12)

and

$$\sigma_{s}=\left(\frac{\Phi_{c}}{\pi d_{0c}^{2}\xi_{0c}}+\frac{\Phi_{m}}{\pi d_{0m}^{2}\xi_{0m}}\right)\overset{5}{\underset{q=1}{\sum }}R_{q}\delta_{q}^{*}\;e_{q}⊗e_{q}$$ (13)

for the *vocalis*. Thus, as an oversimplified representation, the proposed micro-mechanical model can be considered the imbrication of two anisotropic networks of non-linear Zener models embedded in an isotropic hyperelastic matrix (Figure 1): one for the mechanics of fibril bundles and another for their steric interactions. The mechanical response of the *lamina propria* (respectively *vocalis)* depends on 19 (respectively 25) histological and micro-mechanical parameters to be determined:

• *6 (respectively 10) histological parameters*: The fibril’s diameter $d_{0}$ (respectively $d_{0c}$ and $d_{0m}$ ), their waviness amplitude $R_{0}$ (respectively $R_{0c}$ and $R_{0m}$ ), spatial periodicity $H_{0}$ (respectively $H_{0c}$ and $H_{0m}$ ), from which their tortuosity $\xi_{0}$ (respectively $\xi_{0c}$ and $\xi_{0m}$ ) can be estimated, the fibril’s volume fraction Φ (respectively $\Phi_{c}$ and $\Phi_{m}$ ), and initial 3D orientation ( $\theta_{0}$ , $\varphi_{0}$ ). These structural parameters can be determined from histological data.

• *13 (respectively 15) mechanical parameters*: The fibril’s Young’s modulus $E_{f}$ (respectively $E_{fc}$ and $E_{fm}$ ), the matrix shear modulus μ , the transition parameter α (respectively $\alpha_{c}$ and $\alpha_{m}$ ), the elastic interaction coefficients β , κ , and $\delta_{c}$ related to steric effects, and the viscoelastic parameters E , $\eta_{0}$ , ${\dot{\varepsilon }}_{0}$ , and n , along with $E^{\prime }$ , $\eta_{0}^{\prime }$ , and ${\dot{\varepsilon }}_{0}^{\prime }$ .

## 3 Model identification

### 3.1 Experimental database

The relevance of the model was evaluated by comparing its prediction with experimental data from the literature:

• First, to assess the model relevance in the linear viscoelastic regime at small shear strains, we considered data collected by Chan and Rodriguez (2008): “cover” specimens were excised from seven donors (two female and five male donors), between 53 and 88 years old (mean age 67 years). Tissues were collected between 3 and 20 h *post-mortem* before being tested (mean time 10 h). The excised tissues were then subjected to SAOS under physiological conditions (T ≈ 37 ° C, 100 % relative humidity). An oscillatory shear strain $\gamma_{zx}=\gamma_{0}\sin\left(2\pi ft\right)$ was applied in the “longitudinal” plane $\left(e_{z},e_{x}\right)$ , with a prescribed small shear strain amplitude $\gamma_{0}=0.01$ , and a frequency f varied from 1 to 250 Hz. In the following, trends derived from these seven donor-specific covers are represented by an “average target vocal-fold cover,” noted as ${\text{C}}_{\mathrm{SAOS}}$ .

• Second, the model ability to reproduce oscillatory responses in the non-linear regime and finite strains was investigated with respect to data reported by Chan (2018). The author subjected a 60-year-old male “cover” to LAOS with several increasing strain amplitudes $\gamma_{0}=$ [0.05, 0.1, 0.2, 0.5, and 1] along the plane $\left(e_{z},e_{x}\right)$ at a prescribed frequency f= 75 Hz. In the following, the sample chosen as a reference here is noted as ${\text{C}}_{\mathrm{LAOS}}$ .

• Third, the model prediction was compared with vocal-fold layer samples deformed at finite strains and several relevant physiological loadings (*i.e.*, tension, compression, and shear), as reported by Cochereau et al. (2020): two samples of *lamina propria* (covered by the very thin *epithelium* left intact, noted as ${\text{LP}}_{1}$ and ${\text{LP}}_{2}$ ), and two samples of *vocalis* (noted as ${\text{V}}_{1}$ and ${\text{V}}_{2}$ ). As a reminder, each sample was sequentially subjected to longitudinal tension along $e_{z}$ , transverse compression along $e_{x}$ , and longitudinal shear in the plane $\left(e_{z},e_{x}\right)$ . For each loading mode, samples were subjected to 10 load/unload cycles up to Hencky strains $\varepsilon_{zz}^{\max}=$ 0.1, $\varepsilon_{xx}^{\min}=-$ 0.2, and shear $\gamma_{zx}^{\max}=$ 0.6 at constant strain rates $\left|{\dot{\varepsilon }}_{zz}\right|$ , $\left|{\dot{\varepsilon }}_{xx}\right|$ , and $\left|{\dot{\gamma }}_{zx}\right|$ of $\approx 10^{-3}$ ${\text{s}}^{-1}$ .

### 3.2 Optimization procedure

A protocol similar to that adopted by Terzolo et al. (2022) was applied to obtain optimized sets of histo-mechanical parameters:

• For SAOS and LAOS experiments, all histological parameters were initialized and constrained within a corridor of admissible values deduced from the literature, as detailed by Terzolo et al. (2022): 0° $\leq \theta_{0}\leq$ 50°, 20° $\leq \varphi_{0}\leq$ 90°, $10\;\mu m\leq H_{0}\leq 70\;\mu m$ , $1\;\mu m\leq R_{0}\leq 10\;\mu m$ , $10\;nm\leq d_{0}\leq 500\;nm$ , and 0.15≤Φ≤0.55 . For multi-axial experiments achieved with *lamina propria* and *vocalis* samples (Cochereau et al., 2020), we chose the histological parameters already determined by Terzolo et al. (2022), as reported in Table 1.

• For SAOS and LAOS experiments, some of the hyperelastic parameters were constrained within physiological boundaries, *i.e.*, the fibril Young modulus $1\;MPa\leq E_{f}\leq 1\;GPa$ and the matrix shear modulus 1 Pa≤μ≤1.5 MPa . The other parameters, *i.e.*, the transition parameters α and the interaction coefficients β , κ , and $\delta_{c}$ , were left free. It is also important to note that steric interactions are not triggered during simple shear, thus leaving β , κ , and $\delta_{c}$ undetermined for SAOS and LAOS. For the multi-axial experiments performed with *lamina propria* and *vocalis* samples, we considered the hyperelastic parameters determined by Terzolo et al. (2022), except for the shear moduli of the matrices μ , which were explored between 1 Pa and 1 MPa (see comments in the next section).

• The positive viscoelastic parameters, *i.e.*, E , $\eta_{0}$ , ${\dot{\varepsilon }}_{0}$ , and n , along with $E^{\prime }$ , $\eta_{0}^{\prime }$ , and ${\dot{\varepsilon }}_{0}^{\prime }$ , were freely optimized for each of the experiments considered. For SAOS experiments, the power-law exponent n was chosen between 0 and 1 to mimic the recorded shear-thinning behavior (Chan and Rodriguez, 2008). As the LAOS and the multi-axial experiments were performed at a unique strain rate, n could not be determined and was arbitrarily set to the value found for SAOS experiments.

**TABLE 1:** Optimized histological parameters for samples ${\text{C}}_{\mathrm{SAOS}}$ , ${\text{C}}_{\mathrm{LAOS}}$ , ${\text{LP}}_{1}$ , ${\text{LP}}_{2}$ , ${\text{V}}_{1}$ , and ${\text{V}}_{2}$ . Gray-colored columns refer to quantities computed as a function of the determined histological parameters.

Sample	$\theta_{0}\left({}^{\circ}\right)$	$\varphi_{0}\left({}^{\circ}\right)$	$H_{0}\left(\text{μm}\right)$	$R_{0}\left(\text{μm}\right)$	$d_{0}\left(\mu m\right)$	Φ	$\xi_{0}$
${\text{C}}_{\mathrm{SAOS}}$	10.5	83.7	34.5	7.3	0.21	0.30	1.34
${\text{C}}_{\mathrm{LAOS}}$	32.6	65.7	45	4.5	0.23	0.30	1.11
${\text{LP}}_{1}$	16	83	42	5	0.4	0.46	1.13
${\text{LP}}_{2}$	16	83	42.5	5	0.4	0.48	1.13
${\text{V}}_{1c}$	33	70	28	6.4	0.4	0.1	1.4
${\text{V}}_{1m}$	33	70	1,350	130	1	0.7	1.08
${\text{V}}_{2c}$	28	67	30	5.5	0.4	0.12	1.28
${\text{V}}_{2m}$	28	67	1,620	90	1	0.7	1.03

A non-linear constraint optimization process based on a least-squared approach was used to minimize the discrepancies between the model prediction and the experimental macroscale stress–strain curves, as applied by Bailly et al. (2012) and Terzolo et al. (2022). The time integration of the implicit non-linear Maxwell differential Equations 3, 7 was achieved using the *ode15i* solver in ${\text{Matlab}}^{\text{®}}$ (Shampine, 2002).

## 4 Results and discussion

### 4.1 Relevance of histo-mechanical parameters

The set of optimized histological parameters used to reproduce the macroscopic rheological data during SAOS (Chan and Rodriguez, 2008), LAOS (Chan, 2018), and multi-axial loadings (Cochereau et al., 2020) are reported in Table 1. Apart from the remarks already stated by Terzolo et al. (2022) regarding the relevance of these parameters for ${\text{LP}}_{i}$ and ${\text{V}}_{i}$ samples, these values prompt the following comments:

• The optimization led to a collagen content Φ of ≈ 0.47 for ${\text{LP}}_{i}$ samples (*i.e.*, including the *epithelium*, the cover, the intermediate, and the deep layers) versus only ≈ 0.30 for the cover ${\text{C}}_{\mathrm{SAOS}}$ . This finding is consistent with prior experimental evidence, showing that the first sublayer beneath the *epithelium*, *i.e.*, the superficial layer of the *lamina propria* also called “Reinke’s space,” exhibits a fibril content lower than that found in the intermediate and deep layers of the *lamina propria* (Hahn et al., 2006b; Walimbe et al., 2017; Bailly et al., 2018).

• The optimization also yielded a collagen fibril diameter $d_{0}$ close to 200 nm in the cover ${\text{C}}_{\mathrm{SAOS}}$ , against $d_{0}\approx$ 400 nm in the ${\text{LP}}_{i}$ samples. Such a decrease may be explained by the $d_{0}$ -variations reported with the collagen type (Asgari et al., 2017) and with their location across the *lamina propria* (Gray et al., 2000; Tateya et al., 2006; Hahn et al., 2006a; Muñoz-Pinto et al., 2009; Walimbe et al., 2017; Benboujja and Hartnick, 2021). In particular, Muñoz-Pinto et al. (2009) measured that the content of “thin” (respectively “thick”) collagen fibrils decreases (respectively increases) steadily and approximately 10-fold (respectively 15-fold) from the superficial to the deep layers.

• The optimized fibril tortuosity $\xi_{0}$ at rest is ≈ 20 % higher for the ${\text{C}}_{\mathrm{SAOS}}$ experiments than that estimated for the ${\text{LP}}_{i}$ samples. This is consistent with previous observations showing that the intermediate layer of the *lamina propria* is characterized by a dense network of straighter ECM fibrils compared with the superficial and deep layers (Klepacek et al., 2016; Bailly et al., 2018).

• The histological parameters found for the cover sample ${\text{C}}_{\mathrm{LAOS}}$ are very close to the values obtained for the cover sample ${\text{C}}_{\mathrm{SAOS}}$ . The main differences concern the initial fibril orientation ( $\theta_{0}$ and $\varphi_{0}$ ) and tortuosity $\left(\xi_{0}\right)$ . This can be attributed to inter-subject variability.

• The histological parameters of collagen fibrils in the *vocalis* are rather similar than those found for SAOS, LAOS, and ${\text{LP}}_{i}$ samples, except for the fibril content which is much lower. Conversely, the histological parameters of myofibrils are obviously very different.

In addition, the optimized micro-mechanical parameters used to reproduce the macroscopic rheological data during SAOS (Chan and Rodriguez, 2008), LAOS (Chan, 2018), and multi-axial loadings (Cochereau et al., 2020) are reported in Tables 2, 3 for hyperelastic and viscoelastic contributions, respectively. Terzolo et al. (2022) explained the relevance of the hyperelastic parameters in the cases of the ${\text{LP}}_{i}$ and ${\text{V}}_{i}$ samples. In addition, the following remarks can be put forward:

• For the ${\text{LP}}_{i}$ and ${\text{V}}_{i}$ samples, the shear modulus of the matrix μ -coefficient was re-optimized (within physiological boundaries) as the mechanical contribution of the matrix here is related to both the hyperelastic and the viscoelastic contributions (which encompass the fibril/surrounding matrix interactions). Thus, the optimization process led to 200 Pa and 190 Pa for ${\text{LP}}_{1}$ and ${\text{LP}}_{2}$ , respectively, against 330 Pa and 290 Pa in Terzolo et al. (2022), and to 170 Pa for both ${\text{V}}_{i}$ samples, against 900 Pa and 980 Pa.

• As emphasized in Table 2, the matrix shear modulus μ is nearly 10-fold lower for the cover samples ${\text{C}}_{\mathrm{SAOS}}$ and ${\text{C}}_{\mathrm{LAOS}}$ than for the entire ${\text{LP}}_{i}$ samples. The values identified for ${\text{C}}_{\mathrm{SAOS}}$ and ${\text{C}}_{\mathrm{LAOS}}$ are close to the range measured for the elastic shear modulus of hyaluronic acid $\mu_{HA}\approx$ 20–50 Pa [estimated at loading frequencies up to 10 Hz; Heris et al. (2012)], *i.e.*, the most abundant polymer of the ground substance in the *lamina propria.* Hyaluronic acid, known to play a key role in shock absorption during vocal-fold collisions, is found at a higher volume fraction than collagen and elastin in the superficial layer, in contrast to the deep layer (Finck, 2008; Hahn et al., 2006a; Hahn et al., 2006b), which is in line with the identification results (see Table 1). The observed discrepancy in μ -values in Table 2 is probably ascribed to the scarcity of elastin fibrils reported in the superficial layer (and therefore in the cover) in elderly tissues (Roberts et al., 2011).

• The hyperelastic parameters related to the collagen fibril networks are very similar, regardless of the considered samples, *i.e.*, SAOS and LAOS, along with ${\text{LP}}_{i}$ and ${\text{V}}_{i}$ samples. Due to the much softer passive mechanics of myofibrils, their hyperelastic parameters are much lower. Probably for the same reason, the optimized viscoelastic parameters ( E , $\eta_{0}$ , and ${\dot{\varepsilon }}_{0}$ ) found for the *lamina propria*, the SAOS, and the LAOS samples differ by an order of magnitude from those reported for the *vocalis.*

• The viscoelastic parameters of the ${\text{LP}}_{i}$ samples have been identified at a very low strain rate, *i.e.*, close to ${\dot{\varepsilon }}_{0}$ . At this strain rate, the relaxation times $\tau \approx \eta_{0}/E\approx$ 3–15 s are obtained for both vocal-fold layers (similar relaxation times $\tau^{\prime }=\eta_{0}^{\prime }/E^{\prime }$ were found for fibril bundle steric hindrance). It is interesting to note that these results are in line with the rare experimental data available at this scale (Yang, 2008; Shen et al., 2011; Gautieri et al., 2011). For example, Shen et al. (2011) reported typical relaxation times of solvated collagen fibrils in the range of 7–102 s. Moreover, Yang (2008) measured two distinct processes contributing to the stress relaxation of native collagen fibrils immersed in PBS buffer and subjected to 5–7 % strain for 5–10 min: a fast relaxation process with a characteristic time $\tau_{1}\approx 1.8\pm 0.4$ s and a slow relaxation process with $\tau_{2}\approx 60\pm 10$ s. Yang (2008) proposed that $\tau_{1}$ corresponds to the relative sliding of collagen microfibrils, while $\tau_{2}$ refers to the relative sliding of collagen molecules (due to the high level of cross-links between molecules). It is interesting to note that the characteristic times reported for the SAOS and LAOS samples are markedly lower, *i.e.*, $\tau =\eta_{0}/E\approx \;0.42$ s and 0.27 s, respectively. Considering that the model parameters for SAOS and LAOS were determined from experimental data acquired at high frequencies (from 1 to 250 Hz for SAOS and at 75 Hz for LAOS), these low-valued characteristic times are not surprising: additional data at lower strain rates would probably increase these values.

**TABLE 2:** Optimized hyperelastic parameters for samples ${\text{C}}_{\mathrm{SAOS}}$ , ${\text{C}}_{\mathrm{LAOS}}$ , ${\text{LP}}_{1}$ , ${\text{LP}}_{2}$ , ${\text{V}}_{1}$ , and ${\text{V}}_{2}$ .

Sample	$E_{f}\left(MPa\right)$	$\mu \left(Pa\right)$	α	$\beta \left(N\right)$	κ	$\delta_{c}\left(\text{μm}\right)$
${\text{C}}_{\mathrm{SAOS}}$	720	31	1.6 $10^{-3}$	—	—	—
${\text{C}}_{\mathrm{LAOS}}$	720	30	4.6 $10^{-3}$	—	—	—
${\text{LP}}_{1}$	847	200	4.4 $10^{-3}$	2 $10^{-4}$	3	66
${\text{LP}}_{2}$	847	190	4.3 $10^{-3}$	4 $10^{-4}$	3	65.7
${\text{V}}_{1c}$	847	170	4.4 $10^{-3}$	2.2 $10^{-4}$	3	367
${\text{V}}_{1m}$	0.05	170	1.1 $10^{-2}$	2 $10^{-4}$	3	367
${\text{V}}_{2c}$	847	170	4.4 $10^{-3}$	7.6 $10^{-5}$	3	360
${\text{V}}_{2m}$	0.05	170	2.7 $10^{-2}$	7.6 $10^{-5}$	3	360

**TABLE 3:** Optimized viscoelastic parameters for samples ${\text{C}}_{\mathrm{SAOS}}$ , ${\text{C}}_{\mathrm{LAOS}}$ , ${\text{LP}}_{1}$ , ${\text{LP}}_{2}$ , ${\text{V}}_{1}$ , and ${\text{V}}_{2}$ .

Sample	$E\left(MPa\right)$	$\eta_{0}\left(MPa\;s\right)$	$\dot{\varepsilon_{0}}\left(s^{-1}\right)$	n	$E^{\prime }\left(MPa\right)$	$\eta_{0}^{\prime }\left(MPa\;s\right)$	$\dot{\varepsilon_{0}^{\prime }}\left(s^{-1}\right)$
${\text{C}}_{\mathrm{SAOS}}$	3.68	1.56	$2.1\times 10^{-3}$	0.27	—	—	—
${\text{C}}_{\mathrm{LAOS}}$	4.19	1.14	$1.9\times 10^{-3}$	0.27	—	—	—
${\text{LP}}_{1}$	1.47	14.2	$5\times 10^{-4}$	0.27	0.99	8.3	$5.5\times 10^{-3}$
${\text{LP}}_{2}$	1.3	19.6	$6\times 10^{-4}$	0.27	1.63	16	$4.5\times 10^{-3}$
${\text{V}}_{1}$	0.11	0.38	$3.6\times 10^{-3}$	0.27	0.11	0.53	$4.5\times 10^{-3}$
${\text{V}}_{2}$	0.11	1.06	$3.3\times 10^{-3}$	0.27	0.07	0.67	$4.5\times 10^{-3}$

### 4.2 Relevance of the micro-mechanical model for SAOS

**FIGURE 2:**
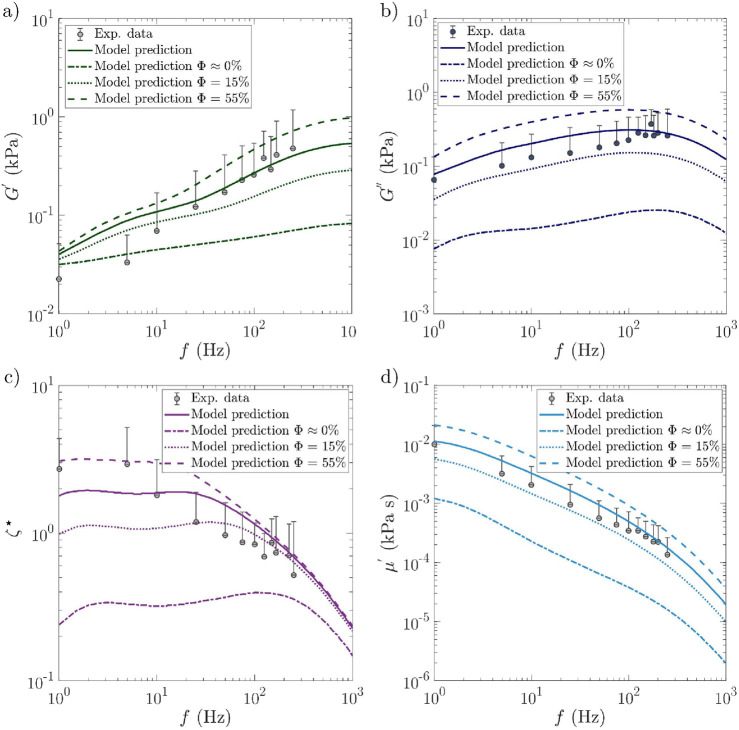
Experimental data (marks) *vs.* macroscale model predictions (lines) obtained for sample ${\text{C}}_{\mathrm{SAOS}}$ : storage $G^{\prime }$ modulus **(a)**, loss G′′ modulus **(b)**, loss factor $\zeta^{⋆}$ **(c)**, and dynamic viscosity $\mu^{\prime }$ **(d)**, as functions of the oscillation frequency f . The continuous line represents the best fit of the model, with the others illustrating the effect of the collagen fibril content Φ . *Source:* experimental data adapted from Chan and Rodriguez (2008). Averaged data and standard deviations from seven human vocal-fold “cover” specimens are reported.

A comparison between the model predictions at macroscale and the SAOS experimental data is provided in Figure 2. In this figure, graphs (a) and (b) show the evolution of the shear storage and loss moduli $G^{\prime }$ and $G^{\prime \prime }$ of sample ${\text{C}}_{\mathrm{SAOS}}$ as functions of the excitation frequency f , whereas graphs (c) and (d) do the same for the loss factor $\zeta^{⋆}=G^{\prime \prime }/G^{\prime }$ and the dynamic viscosity $\mu^{\prime }=G^{\prime \prime }/2\pi f$ , respectively. In these graphs, the model predictions were extended up to f= 1 kHz. Different remarks are highlighted from these graphs:

• For all the rheological functions presented, a fairly good quantitative agreement is obtained between the model predictions (continuous lines) and the experimental data (marks): progressive increase in storage and loss moduli $G^{\prime }$ and $G^{\prime \prime }$ with f up to 200 Hz, power-law decrease in the viscosity $\mu^{\prime }$ , and Carreau-like evolution of the loss factor $\zeta^{⋆}$ , with a marked power-law reduction above 10–50 Hz.

• More particularly, it is interesting to note that the model nicely predicts the experimental “cross-over” zone at approximately 50–100 Hz, *i.e.*, the zone within which (i) the storage modulus $G^{\prime }$ switches from lower to higher than the loss modulus $G^{\prime \prime }$ and (ii) the loss factor $\zeta^{⋆}$ switches from constant to remarkable decrease. This transition zone also coincides with that where some issues occur during vocal-fold vibration in human phonation. For fold vibration at low frequencies, *i.e.*, below 50–100 Hz, viscous effects dominate $\left(G^{\prime \prime }\geq G^{\prime }\right)$ so that this should give rise to critical tissue overdamping, preventing proper periodic oscillations of vocal folds. In contrast, the dominant elastic properties at higher frequencies should restrain tissue damping (see the power-law decrease in the loss factor in Figure 2c), thus allowing the occurrence of proper periodic motion during vocal-fold vibration (Chan and Rodriguez, 2008).

• To illustrate the role of histological parameters on the rheological response of SAOS samples, we have reported two additional discontinuous lines in Figure 2. These trends emphasize the effects induced by variations in the volume fraction of collagen fibrils Φ (here, Φ was chosen due to its wide variations between individuals but also within the vocal-fold layers themselves): the case where Φ= 0.15 and the case where Φ= 0.55, *i.e.*, the minimum and maximum values found in the literature for the *lamina propria.* As shown in Figure 2, when Φ varies in the physiological corridor, the qualitative trends are preserved for all viscoelastic properties ( $G^{\prime }$ , $G^{\prime \prime }$ , $\zeta^{⋆}$ , and $\mu^{\prime }$ ). However, the higher the fibril content, the higher the rheological functions, albeit with (i) marked differences (for $G^{\prime }$ at high frequencies, for $\zeta^{⋆}$ at low frequencies, *e.g.*, for $G^{\prime \prime }$ , and $\mu^{\prime }$ at all frequencies) and (ii) a slight shift of the cross-over zone toward lower frequencies as Φ is increased. Note that the case of Φ≈ 0 was also predicted in Figure 2 as a theoretical extreme case (not physiological), assuming a quasi-total absence of collagen fibers in the vocal-fold cover, which would thus become close to a homogeneous, isotropic neo-Hookean material with the same mechanical properties as the matrix alone. These simulations clearly emphasize the major mechanical role played by the collagen fibrous network, along with its interaction with the surrounding ground substance, in response to the oscillatory shear of the vocal-fold cover.

### 4.3 Relevance of the micro-mechanical model for LAOS

**FIGURE 3:**
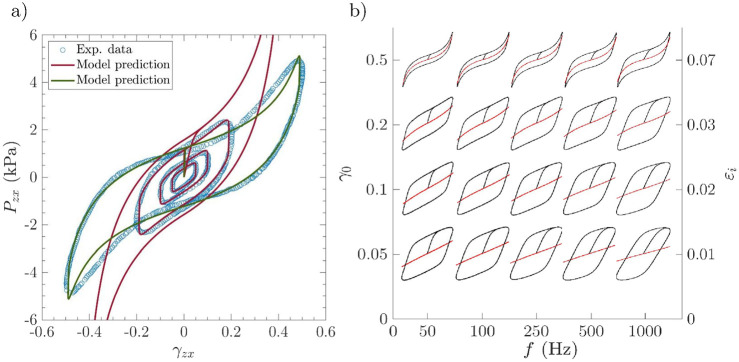
LAOS results: **(a)** macroscale stress–strain data *vs.* model predictions obtained for sample ${\text{C}}_{\mathrm{LAOS}}$ tested at f = 75 Hz and with $\gamma_{0}$ varied from 0.05 to 0.5. *Source:* experimental data adapted from Chan (2018). **(b)** Predicted Lissajous stress–strain curves plotted in the Pipkin space $\left\{f,\gamma_{0}\right\}$ or $\left\{f,\varepsilon_{i}^{\max}\right\}$ , where $\varepsilon_{i}^{\max}$ is the maximal cyclic tensile strain the fibrils are subjected to. Black solid lines represent the total stress, while red solid lines represent neutral contribution.

In Figure 3a, we have reported a collection of Lissajous stress–strain curves predicted by the model. These curves are compared with those in LAOS experiments obtained at a frequency f = 75 Hz and cyclic amplitudes $\gamma_{0}$ varied from 0.05 to 0.5. In addition, Figure 3b presents a series of normalized Lissajous stress–strain curves predicted by the model in the Pipkin space $\left\{f,\gamma_{0}\right\}$ or $\left\{f,\varepsilon_{i}^{\max}\right\}$ ( $\varepsilon_{i}^{\max}$ is the maximal cyclic tensile strain the fibrils are subjected to), when f and $\gamma_{0}$ are varied from 50 Hz to 1 kHz and from 0.05 to 0.5, respectively (Ewoldt et al., 2008; Chan, 2018). Within each contour plot, the black line represents the total visco-hyperelastic stress, whereas the red line is the hyperelastic or neutral stress contribution. Different trends can be highlighted.

• *Influence of the strain amplitude* $\gamma_{0}$ : Figure 3a shows a strong quantitative agreement between the model predictions (red line) and the experimental data at stabilized cycles when $\gamma_{0}\;\leq$ 0.2. In particular, the model can capture the strong non-linear response of the tested sample, with, in particular, a proper modeling of the stress hysteresis induced by viscoelastic effects. In addition, the cyclic stress–strain curves progressively deviate from a linear strain hardening at low shear strain amplitudes ( $\gamma_{0}\leq$ 0.1), which corresponds to the initial linear (un)folding of collagen fibril at small strains, toward a marked non-linear strain-hardening at higher strain magnitudes (in J-shape), where the non-linear hyperelastic stretching of collagen fibrils is triggered. This trend is also fairly well illustrated by the neutral stress responses of the Pipkin diagram shown in Figure 3b. This diagram also proves that the trend is preserved independently of the cycling frequency. Finally, it is worth noting from Figure 3a that the predicted strain-hardening at the highest strain magnitude $\gamma_{0}=0.5$ largely overestimates the cycle observed experimentally. Presumably, during the experiments, the tested cover exhibited a Mullins-like effect, as often observed in elastomers, gels, and soft living tissues (Diani et al., 2009; Peña et al., 2009; Rebouah et al., 2017; Rebouah and Chagnon, 2014; Zhan et al., 2024). This could yield to a stress softening of their mechanical behavior upon cycling. The Mullins effect can be caused by a number of irreversible mechanisms, *e.g.,* the rupture of physical or covalent cross-links and the possible disentanglement of molecular chains*.* These mechanisms are not taken into account in the current micro-mechanical model. Yet, a possible way to account for these phenomena would consist in altering, with proper kinetics, the histo-mechanical properties of the collagen fibrils, such as their modulus $E_{f}$ (to account for damage) and their initial length $\ell_{0}^{f}$ or tortuosity $\xi_{0}$ (to account for disentanglement). In support of this hypothesis to be explored in future work, Figure 3a shows that lowering (respectively increasing) $E_{f}$ (respectively $\xi_{0}$ ) from 720 MPa to 400 MPa (respectively from 1.11 to 1.12) would lead to a more appropriate prediction of the experimental stress–strain curve performed at $\gamma_{0}=0.5$ (see green line).

• *Influence of the loading frequency* f : As shown in Figure 3b, the loading frequency f markedly alters the Lissajous curves. Regardless of the strain magnitude $\gamma_{0}$ , the higher the f -value, the higher the stress levels and the stress hysteresis. These trends are in qualitative agreement with measurements acquired on other vocal-fold cover samples (Chan, 2018).

### 4.4 Relevance of the micro-mechanical model for finite strain multi-axial cyclic loadings

**FIGURE 4:**
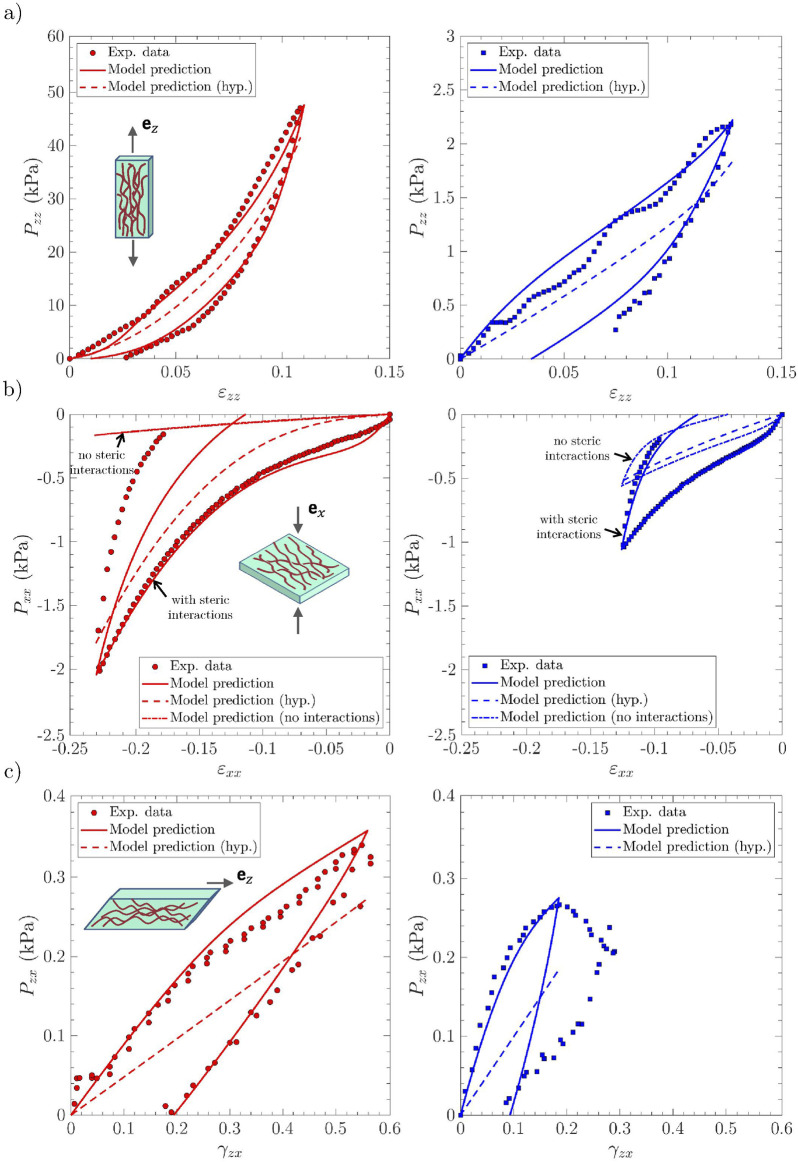
Macroscopic viscoelastic stress–strain curves of vocal-fold sublayers under multi-axial cyclic loadings. Experimental data *vs.* model predictions obtained for the *lamina propria* sample ${\text{LP}}_{1}$ (*left*, in red) and the *vocalis* sample ${\text{V}}_{1}$ (*right*, in blue): **(a)** longitudinal tension; **(b)** transverse compression; **(c)** longitudinal shear. Several model predictions are compared: the visco-hyperelastic model with steric interactions between fibril bundle (*solid lines*); the non-viscous hyperelastic model previously described by Terzolo et al. (2022) (noted ‘hyp.,’ *dashed lines*); the visco-hyperelastic model albeit with no steric interactions between fibril bundle (*dashed*–*dotted lines*) for compression loading solely (panel b).

**FIGURE 5:**
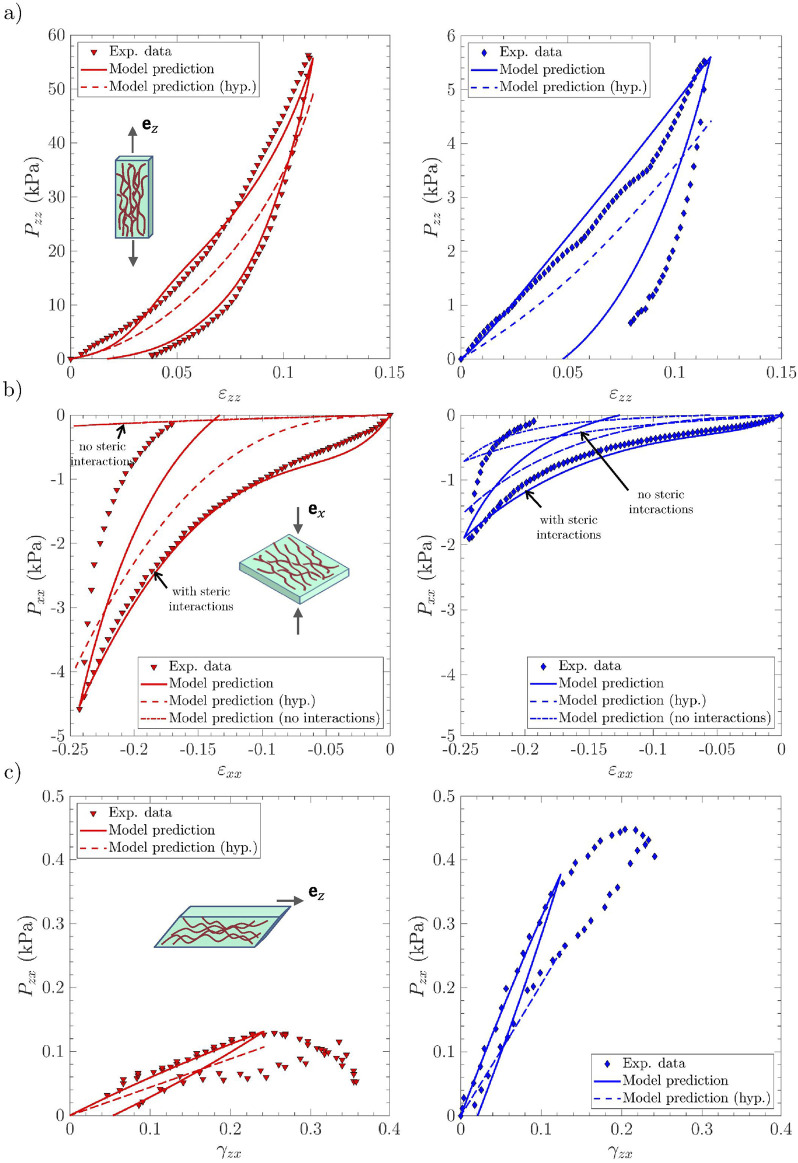
Same as Figure 4 for samples ${\text{LP}}_{2}$ (*left*, in red) and ${\text{V}}_{2}$ (*right*, in blue).

**FIGURE 6:**
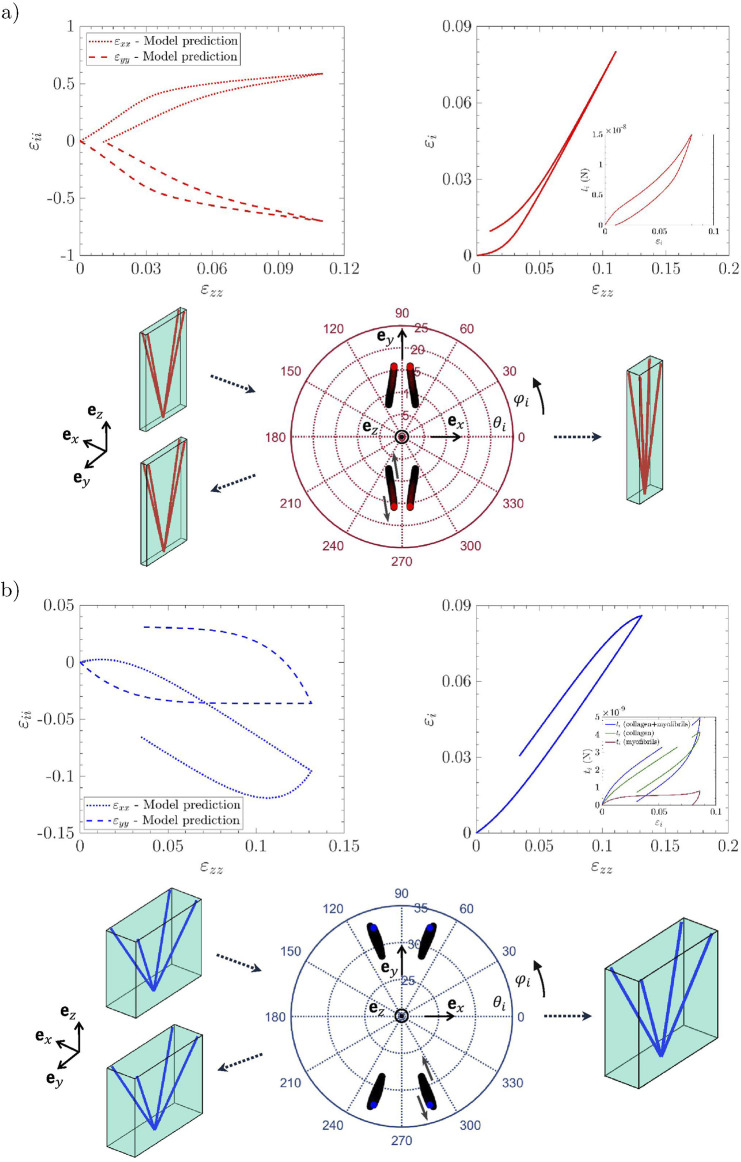
Evolution of multiscale descriptors for the *lamina propria* ${\text{LP}}_{1}$ **(a)** and the *vocalis* ${\text{V}}_{1}$ **(b)** during tension along $e_{z}$ : *(top left)* macroscopic strain paths; *(bottom)* stereographic projection of the four orientation vectors $e_{i}$ from initial to final state; *(top right)* tensile strain of the fibril chord $\varepsilon_{i}$ and corresponding tension $t_{i}$ .

**FIGURE 7:**
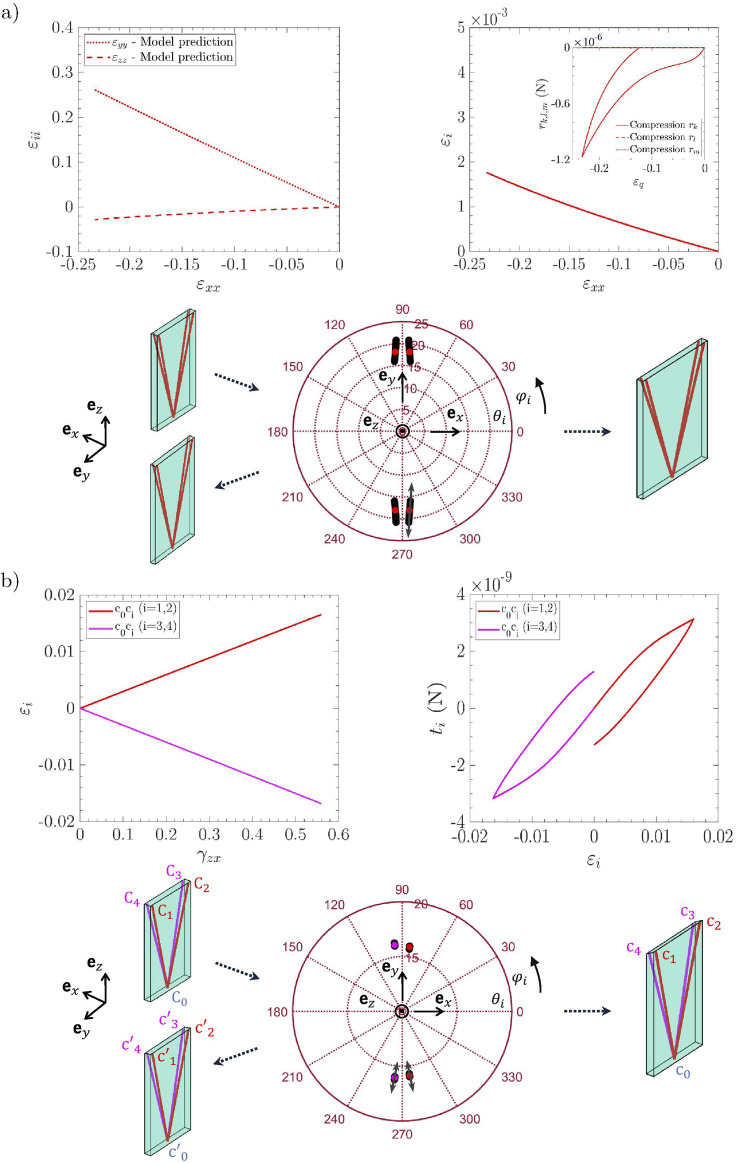
Evolution of multiscale descriptors for the *lamina propria* sample ${\text{LP}}_{1}$ during compression along $e_{x}$ **(a)**, and shear in the plane ( $e_{z}$ and $e_{x}$ ) **(b)**. Compression case: *(top left)* macroscopic strain paths; *(bottom)* stereographic projection of the four orientation vectors $e_{i}$ from initial to final state; *(top right)* tensile strain of the fibril chord $\varepsilon_{i}$ and interaction forces $R_{q}$ . Shear case: *(top left)* tensile strain of the fibril chord $\varepsilon_{i}$ ; *(top right)* corresponding tension $t_{i}$ ; *(bottom)* same as in **(a)**.

Macroscopic stress–strain predictions are compared with the reference experimental data in Figure 4 (respectively Figure 5), for the *lamina propria* and *vocalis* samples ${\text{LP}}_{1}$ and ${\text{V}}_{1}$ (respectively ${\text{LP}}_{2}$ and ${\text{V}}_{2}$ ), and the three cyclic loadings these samples were subjected to, *i.e.*, longitudinal tension, transverse compression, and longitudinal shear. For each case, the “neutral” curve already predicted by Terzolo et al. (2022) was reported (see dotted lines). The strain-induced evolution of micro-mechanical descriptors during cyclic tension is displayed in Figure 6 (an illustrative case of ${\text{LP}}_{1}$ and ${\text{V}}_{1}$ ), with compression and shear results summarized in Figure 7 (an illustrative case of ${\text{LP}}_{1}$ ).

The results for the first loading cycle are discussed below for each loading mode:

• *Longitudinal tension*: The model prediction for longitudinal tension along $e_{z}$ is fairly good for both the *lamina propria* and the *vocalis* samples, as emphasized in Figures 4a–5a. In particular, compared with the hyperelastic formulation proposed by Terzolo et al. (2022), *i.e.*, the neutral curves, the model can now capture the stress hysteresis and the residual strains after unloading. These tendencies are inherited from microscale viscoelastic effects, together with the rearrangement of the tissue microstructures. This is illustrated in Figure 6 and Supplementary Figure S1, in which one can assess the irreversible unfolding and rotation of fibrils that are predicted during cyclic tension, both for the *lamina propria* and the *vocalis* samples. It is interesting to note that the predicted stress hysteresis and residual strain of collagen fibrils were experimentally observed by Yang (2008). For the *vocalis*, the predicted tensions in both collagen fibrils, $t_{ic}$ , and myofibrils, $t_{im}$ , are plotted in the inset of Figure 6b. Although the key role played by the sheaths of collagen fibers surrounding muscle fibers in the passive tensile properties of the tissue was already evidenced during monotonic loading, the strong contribution of myofibrils to inelastic effects and residual strains after unloading is clearly highlighted here.

• *Transverse compression*: Figures 4b, 5b prove that the model predictions are also in good agreement with the experimental data recorded during transverse compression. Moreover, as already pointed out by Terzolo et al. (2022), steric interactions are of major importance for the *lamina propria* and the *vocalis* mechanics during compression. This characteristic is preserved with the visco-hyperelastic formulation: if steric hindrance effects are deactivated in the model (see model predictions with “no steric interactions” in Figures 4b, 5b, dash-dotted lines), the deformation of the visco-hyperelastic fibril bundles is not sufficient to capture the *lamina propria* stress hysteresis and residual strain experimentally observed. Thus, fibril bundle repulsion forces $R_{q}$ and their viscoelastic contributions $R_{q}^{ve}$ appear to be of critical importance to properly reproduce the compression behavior of both vocal-fold layers (see model predictions with “steric interactions” in Figures 4b, 5b, solid lines). No other significant microscale deformation mechanisms (such as rotation or noticeable unfolding of fibrils) were predicted under transverse compression (Figure 7a).

• *Longitudinal shear*: The mechanical contribution of the matrix plays a major role in the overall shear response of the *lamina propria* and the *vocalis*, as already stressed by Terzolo et al. (2022). On this basis, the fibril viscoelastic properties and interactions with the surrounding ground substance allowed, via the microscopic tension $t_{i}$ (Figure 7b), to satisfactorily reproduce the experimental trends observed during the load/unload sequence at the tissue scale (Figures 4c–5c).

**FIGURE 8:**
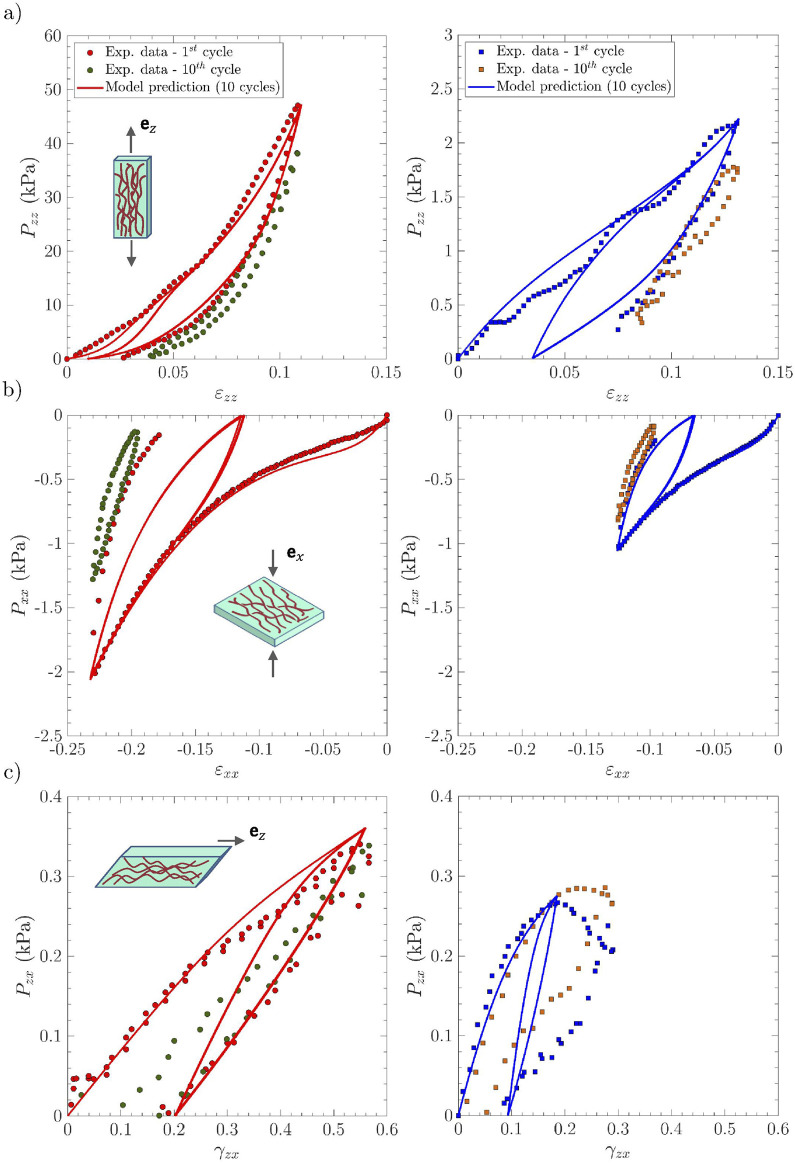
Same as in Figure 4, albeit for 10 cycles: experimental data *vs.* model predictions. The experimental 10th cycle is displayed in green symbols for the *lamina propria* sample ${\text{LP}}_{1}$ *(left)* and in orange symbols for the *vocalis* sample ${\text{V}}_{1}$ *(right)*. Experimental intermediate cycles are not reported for the sake of clarity.

Finally, the relevance of the visco-hyperelastic model to simulate the sequential series of 10 load–unload cycles and the tissue response as a function of load history is assessed. Figure 8 shows the comparison of the theoretical predictions with the reference cyclic data for the three loading modes. If the decrease in stress hysteresis is qualitatively well captured by the model once the first cycle has been completed in tension, compression, and shear, the predictions fail to simulate the progressive decrease in peak stresses measured after repeated loading paths, along with the increase in residual strains after repeated unloading paths, which are particularly observed in tension and compression. According to the model, a steady state is reached practically after the first load/unload sequence, whereas the stabilized behavior is only really observed experimentally after the $5^{\text{th}}$ cycle (or even up to the $10^{\text{th}}$ cycle, depending on the sample and loading mode). As mentioned for LAOS results, these accommodation behaviors resemble Mullins-like effects, which are not taken into account in the present formulation of the model.

### 4.5 Relevance of the model for predicting future patho/physiological variations and assisting biomedical developments

The micro-mechanical model developed in this work has been calibrated to reproduce the microstructural specificities and multiscale behavior of healthy human vocal-fold tissues, combining a wide range of histo-mechanical measurements collected from the available literature. By adjusting these input data, it could be adapted without major difficulties to predict the mechanics of pathological human vocal tissues (Hantzakos et al., 2009; Finck, 2008), animal vocal tissues (Li et al., 2024), or (bio)composites developed to replace/reconstruct the fibrous architecture and vibro-mechanical performance of the vocal folds after surgery (Heris et al., 2012; Li et al., 2016; Jiang et al., 2019; Latifi et al., 2018; Ravanbakhsh et al., 2019; Ferri-Angulo et al., 2023).

The model could also be used to predict the evolution of the mechanical properties of the same tissue following an alteration in its microstructural arrangement, due, for example, to (i) its growth and remodeling with age [by simulating, *e.g.*, a progressive decrease in the volume fraction of elastin, an increase in that of collagen, and muscle atrophy (Roberts et al., 2011; Kuhn, 2014; Li et al., 2024)], (ii) scarring lesions [by simulating fibrosis and an increase in the collagen content, along with changes in fibril tortuosity compared to the healthy case (Heris et al., 2015; Li et al., 2016)], (iii) the appearance of a lesion following phonotrauma [by simulating damage mechanisms likely to occur at the fibril’s level (Miller and Gasser, 2022)], or (iv) a therapeutic treatment [by simulating the addition of a soft hydrogel to the matrix composite, for example (Li et al., 2016; Mora-Navarro et al., 2026)].

To better understand the impact of these histological variations on vocal-fold vibrations at the larynx level (in the case of native tissue but also injured, repaired, or replaced tissue), this original constitutive law should be implemented in a finite element code reproducing the vocal folds in their 3D anatomical geometry, as in current 3D phonation models (Döllinger et al., 2023). In doing so, microstructure-based simulations could improve the currently limited knowledge of the links between the specific microarchitecture of the vocal folds and their unique macroscale vibratory performance. Moreover, they should guide the design of fiber-reinforced biomaterials currently under development for functional voice restoration.

## 5 Conclusion

A better understanding of human phonation requires an in-depth study of the viscoelastic properties of vocal folds. To this end, this study proposes to enrich a recent 3D micro-mechanical model of vocal-fold tissues, which was hitherto capable of predicting their non-linear, elastic, and anisotropic mechanical behavior at various spatial scales (micro to macro) (Terzolo et al., 2022). This was achieved by adding viscoelastic mechanisms at the scale of their collagen fibril and myofibril bundles. These improvements now enable the model to capture the viscoelastic properties of vocal-fold tissues from small to finite strains, such as their non-linear strain-rate sensitivity—on which their damping and oscillation onset properties strongly depend; their stress-hysteretic response, and the inelastic deformations typically measured during cyclic loading. In addition, the model allows the microstructural rearrangements to be predicted, which are often very challenging to identify experimentally.

This model was successfully used to reproduce various sets of *ex vivo* data available in the literature and complement them with original theoretical data, providing specific micro-mechanism scenarios for each. This identification was carried out for a wide variety of loading conditions at different rates: low-frequency cyclic tension, compression, and shear in large deformations; and high-frequency oscillatory shear from small to large deformations (SAOS for the linear viscoelasticity regime and LAOS for the non-linear viscoelasticity regime). The model predictions are in quantitative agreement with macroscopic experimental trends and clearly highlight the key impact of microscopic histo-mechanical descriptors on vocal-fold dynamics, such as the volume fraction of collagen fibrils in the cover, their tortuosity at rest, their mechanics, and their interactions. This micro-mechanical model can be implemented in finite element codes to further simulate the transient dynamics of vocal folds with relevant histo-mechanical properties.

However, some model limitations should be improved. For example, coarse-grained atomistic/molecular simulations would probably provide relevant information to strengthen the physical links between the time-dependent nanostructural rearrangements and the phenomenological approach proposed herein at the fibril scale. Furthermore, the model does not allow the Mullins-like effects commonly observed in vocal tissues to be adequately described: combined with additional experiments focused on this aspect, the model could be improved based on formulations proposed for other materials, such as structured elastomers (Rebouah and Chagnon, 2014; Rebouah et al., 2017).

## Data Availability

The raw data supporting the conclusions of this article will be made available by the authors, without undue reservation.
