# Supplementary material for: A fibril-scale visco-hyperelastic model for the mechanics of vocal-fold tissues
Source: Front Bioeng Biotechnol. 2026 Jan 5;13:1670567. doi: 10.3389/fbioe.2025.1670567 (PMC12813094; doi:10.3389/fbioe.2025.1670567)
Supplement: Supplementary file 1 [file DataSheet1.pdf]

Supplementary Material

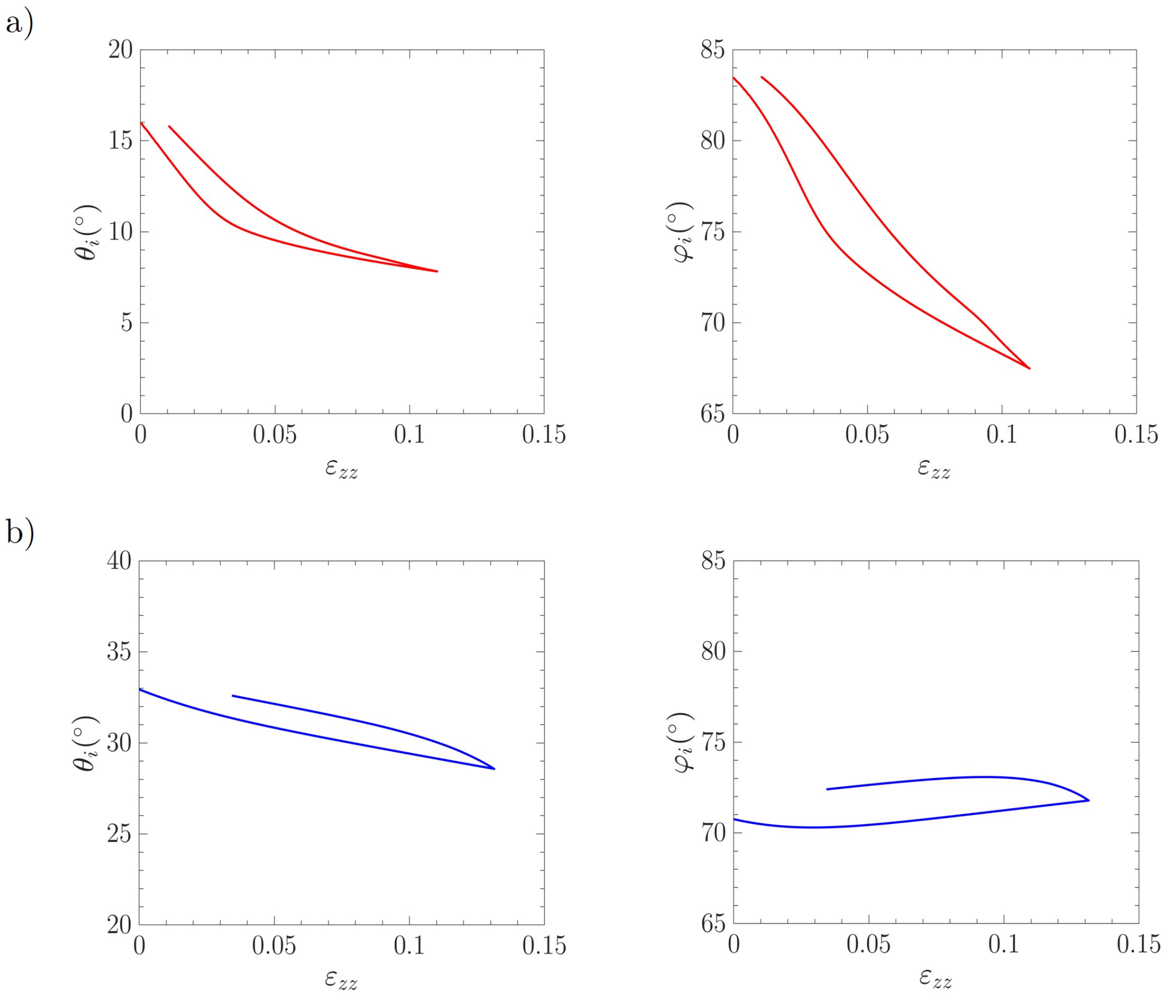

Figure S1. Viscoelastic strain-induced evolution of the orientation vectors $\mathbf{e}_i$ from initial to final state, $i \in [1..4]$ during tension along $\mathbf{e}_z$: a) *lamina propria* LP_1 (in red); b) *vocalis* V_1 (in blue).
